# Role of Caspases and Gasdermin A during HSV-1 Infection in Mice

**DOI:** 10.3390/v14092034

**Published:** 2022-09-13

**Authors:** Lupeng Li, Stephen B. Kovacs, Ine Jørgensen, Heather N. Larson, Helen M. Lazear, Edward A. Miao

**Affiliations:** 1 Department of Immunology, Duke University School of Medicine, Durham, NC 27710, USA; 2 Department of Molecular Genetics and Microbiology, Duke University School of Medicine, Durham, NC 27710, USA; 3 Department of Microbiology and Immunology, University of North Carolina at Chapel Hill, Chapel Hill, NC 27599, USA

**Keywords:** HSV-1, axon pruning, caspase-6, caspases, ASC, gasdermin A, keratinocyte, cell death, mice

## Abstract

Herpes simplex virus type 1 (HSV-1) infection can manifest locally as mucocutaneous lesions or keratitis and can also spread to the central nervous system to cause encephalitis. HSV-1 establishes a lifelong latent infection and neither cure nor vaccine is currently available. The innate immune response is the first line of defense against infection. Caspases and gasdermins are important components of innate immunity. Caspases are a family of cysteine proteases, most of which mediate regulated cell death. Gasdermins are a family of pore-forming proteins that trigger lytic cell death. To determine whether caspases or gasdermins contribute to innate immune defenses against HSV-1, we screened mice deficient in specific cell death genes. Our results indicate a modest role for caspase-6 in defense against HSV-1. Further, *Asc*^–/–^*Casp1/11*^–/–^ mice also had a modest increased susceptibility to HSV-1 infection. Caspase-7, -8, and -14 did not have a notable role in controlling HSV-1 infection. We generated *Gsdma1-Gsdma2-Gsdma3* triple knockout mice, which also had normal susceptibility to HSV-1. We confirmed that the previously published importance of RIPK3 during systemic HSV-1 infection also holds true during skin infection. Overall, our data highlight that as a successful pathogen, HSV-1 has multiple ways to evade host innate immune responses.

## 1. Introduction

Herpes simplex virus type 1 (HSV-1) is a double-stranded DNA herpesvirus [1]. HSV-1 is prevalent in human populations, with over two-thirds of the world population infected [2]. In the United States, HSV-1 causes lifelong infection in most adults [3]. Primary HSV-1 infection typically begins with the infection of epithelial cells (e.g., keratinocytes), followed by innervating sensory neurons [4]. HSV-1 often establishes latency in sensory ganglia and can later reactivate when the host becomes stressed. HSV-1 infection manifests as orolabial herpes, corneal infection, skin infection, and more severely, central nervous system (CNS) infection. 

Several murine models are routinely used for HSV-1 pathogenesis studies. A mouse flank scarification model recapitulates the infection cycle of HSV-1 in humans [5]. In a flank scarification model [6,7,8], HSV-1 is applied topically onto the depilated flank skin where it replicates and spreads in keratinocytes before it infects innervating sensory neurons in the skin. HSV then traffics via the axon to neuron cell bodies located in dorsal root ganglia (DRG), where it replicates and spreads to other neurons within the DRG. HSV-1 then traffics back through the axons to an entire dermatome, forming zosteriform skin lesions. While HSV-1 is typically a pathogen of the peripheral nervous system, the virus also can invade the CNS, producing an ascending infection from the DRG into the spinal cord and brain. In a footpad infection model, neuroinvasive viruses infect epithelial cells and spread through the sciatic nerve to the CNS [9,10]. HSV-1 can also cause the infection of the eye in humans and is modeled in mice using a corneal scarification model in which HSV-1 first infects the corneal epithelium and then traffics via the ciliary and ophthalmic nerves to the trigeminal ganglion (TG) and brain [11,12]. 

As a successful host-adapted pathogen, HSV-1 has evolved multiple immune evasion strategies [1]. However, HSV-1 can be recognized by innate immunity, which is the first line of defense against invading pathogens [1,13]. Regulated cell death, such as apoptosis, is important for clearing infection by removing replicative niches and releasing danger signals to recruit immune cells [14,15]. Caspases are a group of cysteine-aspartic proteases that are involved in multiple forms of cell death. Based on sequence similarity and function, caspases are divided into two groups: apoptotic caspases (caspase-8, -9, -2, -3, -6, and -7) and inflammatory caspases (caspase-1 as well as -4/5 in humans and -11 in mice). However, these two groups have become less distinct in recent years. For example, caspase-3 can also trigger pyroptosis in certain cell types [16], and we recently proposed that caspase-7 is functional in both apoptotic and inflammatory caspase signaling [17]. Another member of the caspase family, caspase-14, is specifically expressed in keratinocytes [18,19]. Although caspase-14 knockout (*Casp14*^–/–^) mice have visually normal skin and fur in our colony, caspase-14 does have regulatory effects on keratinocyte differentiation [20]. 

Apoptosis is a “silent” form of cell death activated by numerous internal or external signals during normal development processes and under stress. Apoptosis is initiated and executed by apoptotic caspases, primarily by caspase-3, which upon activation is sufficient to accomplish the processes of converting a live cell into apoptotic bodies. The apoptotic caspases are broadly expressed in most cell types. Apoptosis is important in antiviral host defense by removing infected cells [14]. HSV-1 induces apoptosis and sometimes blocks apoptosis from killing infected cells [21,22,23]. 

In contrast to apoptosis, pyroptosis is a form of regulated lytic cell death mediated by gasdermins (GSDM) [24]. The best-characterized gasdermin, GSDMD, induces pyroptosis after cleavage by inflammatory caspases, caspase-1 or -11 [25,26,27]. Another member of the gasdermin family, GSDMA, is expressed primarily in keratinocytes and other stratified epithelia, similar to caspase-14 [28]. GSDMA can be cleaved and activated by the *Streptococcus* protease SpeB during infection to trigger pyroptosis of keratinocytes [29,30]. GSDMA is also implicated in apoptosis [31] and autophagy [32]. 

We assessed the role of caspases-1/11, -6, -7, -8, -14, the adaptor protein ASC involved in the mediating pyroptosis upstream of GSDMD, and GSDMA using various HSV-1 infection models.

## 2. Materials and Methods

### 2.1. Mice

Mice were housed in a specific-pathogen-free facility at the University of North Carolina (UNC) at Chapel Hill or Duke University: wild-type C57BL/6 (Jackson, Bar Harbor, ME, USA 0006664), *Casp6*^–/–^ (Jackson 006236), *Casp7*^–/–^ (Jackson 006237), *Rag1*^–/–^ (Jackson 002216), *Casp8*^–/–^*Ripk3*^–/–^ (ref. [33]), *Casp14*^–/–^ (ref. [34]). *Asc*^–/–^*Casp1/11*^–/–^ mice were generated by crossing *Asc*^–/–^ (ref. [35]) with *Casp1/11*^–/–^ (ref. [36]) in house. *Gsdma*^–/–^ mice were generated by the UNC Animal Model Core using CRISPR. *Gsdma*^–/–^ mice appear healthy and breed well. The following primers were used to genotype *Gsdma*^–/–^ mice: WT = 207 bp (WT F 5′-CTG CTG AAC AGG ACC TAG CAT-3′, WT R 5′-ACT CAA AAG TTG CCA CTC TTC TC-3′); *Gsdma*^–/–^ = 532 bp (Gsdma-5ScF1 5′-TGC TCC TAC AGA TGC TCG GTC-3′, Gsdma-3ScR1 5′-CAT CTA TAC TCC AGT TCC CTC CAG-3′). Animal protocols were approved by the Institutional Animal Care and Use Committee (IACUC) at UNC-Chapel Hill or Duke University and met the US National Institutes of Health guidelines.

### 2.2. Cells and Viruses

Vero cells (African green monkey kidney epithelial cells) were obtained from Dr. Raphael H. Valdivia (Duke University) and cultured in Dulbecco’s modified Eagle’s medium (DMEM, Gibco) supplemented with 20 mM HEPES (Gibco), 10% fetal bovine serum (Sigma-Aldrich, St. Louis, MO, USA), and 100 I.U./mL Penicillin-Streptomycin (Gibco). HSV-1 strain NS, a clinical isolate, was described previously [37]. WT HSV-1 strain F and HSV-1 Δ*Us3* (R7041) were described previously and obtained from Dr. David Bloom (University of Florida) [38]. HSV-1 viral stock was made by infecting Vero cells and collecting supernatant and infected Vero cells. Viral titer was determined by plaque assay on Vero cells [39].

### 2.3. Mouse Flank Scarification Model

The HSV-1 flank infection was performed as described [7,39,40] with slight modifications. Briefly, one day before infection, mice were anesthetized with isoflurane (Dechra Pharmaceuticals, Northwich, UK). The right flanks of the mice were shaved, and then chemically depilated using Nair cream (Church and Dwight, Ewing Township, NJ, USA). Nair was wiped off and the right flank was flushed with sterile water to remove residual Nair. Twenty-four hours later, the mice were anesthetized, and 2 × 10^7^ PFU/mL of WT HSV-1 or 2 × 10^8^ PFU/mL of HSV-1 Δ*Us3* strain in a volume of 5 µL was applied onto the skin, a 27 G needle (BD PrecisionGlide, Franklin Lakes, NJ, USA) was used to gently scratch the skin 40 times within an area of 4 mm^2^. The infected mice were anesthetized for 10 more minutes to allow the viral suspension to dry. The skin lesion severity was recorded from 3 to 10 days post-infection (dpi) as below: 0, no lesions; 1, discrete pinprick lesions; 2, coalesced lesions; 3, patches of confluent lesions; and 4, complete dermatome. Survival was monitored at least until 21 dpi before mice were euthanized. 

### 2.4. Mouse Footpad Model

The HSV-1 footpad infection model was performed as described [41]. Briefly, mice were anesthetized with isoflurane. The right hind footpad was injected with 3.3 × 10^5^ PFU/mL of HSV-1 in a volume of 30 µL subcutaneously with a 30 G needle (BD PrecisionGlide). Note that different numbers of PFU are injected in the different routes because each route has a different efficiency of infection.

### 2.5. Mouse Corneal Model

The HSV-1 corneal infection was performed as described [42]. Briefly, mice were anesthetized with isoflurane. Then, the right eyes of mice were gently scarified with a 30 G needle 20 times, and 2 × 10^8^ PFU/mL of HSV-1 in a volume of 5 µL was applied to the eye. The eyelids were massaged 5 times to facilitate the absorbance of the viral suspension. The mice were anesthetized for another 10 minutes before returning to their home cage. Survival was monitored daily at least until 21 dpi.

### 2.6. Viral Titers

Viral titers were determined by plaque assay on Vero cells [39] or by quantitative PCR (qPCR). For plaque assays, tissues were removed following euthanasia and cut with scissors and ground with a pestle. The processed tissues were resuspended in DMEM and titered on Vero cells [39]. For qPCR, DNA was extracted from mouse tissues using DNeasy Blood & Tissue Kits (QIAGEN, Hilden, Germany). PCRs were carried out using 2X SYBR Green Master Mix (Bio-Rad, Hercules, CA, USA) with primers targeting the DNA polymerase gene of HSV-1 (5′-ATC AAC TTC GAC TGG CCC TT-3′ and 5′-CCG TAC ATG TCG ATG TTC AC-3′) [43,44,45]. A standard curve of 1 to 10^7^ copies of HSV-1 DNA was used to determine the viral titers. PCR reactions were performed with the Quantstudio Real-Time PCR Systems (Thermo Fisher Scientific, Waltham, MA, USA).

### 2.7. Western Blot

Western blot was performed as described [17]. After euthanasia, both ears of each mouse were removed and placed in liquid nitrogen for grinding. The ground powder was resuspended in 200 µL of PBS plus protease inhibitors (Sigma-Aldrich, St. Louis, MO, USA; Cat#11836153001). The suspension was then centrifuged, and the supernatant was aspirated for Western blot. The pellet after centrifugation was resuspended with 200 µL of RIPA buffer (Thermo Fisher Scientific) plus protease inhibitor. The resuspension was centrifuged again, and the supernatant was aspirated for Western blot. Protein concentrations were normalized based on buffer (PBS or RIPA buffer) using the BCA Protein Assay Kit (Thermo Fisher Scientific). The anti-GSDMA antibody was purchased from Santa Cruz Biotechnology (Dallas, TX, USA; Cat# sc-376318).

### 2.8. Histological Staining

H&E staining was performed as described previously [17]. After euthanasia, the ears of naïve mice were removed and placed in 10% Neutral Buffered Formalin (VWR International, Radnor, PA, USA). Fixed tissues were submitted to UNC Cell Services and Histology Core for embedding, cutting, and H&E staining.

### 2.9. Statistics

The results are expressed as scatter dot plots with bars representing the median. Statistical analysis was performed by two-sided Mann-Whitney *U*-test, one-way ANOVA with post-hoc test, or log-rank Mantel-Cox test using the GraphPad Prism v8 software (San Diego, CA, USA). *p <* 0.05 was considered significant.

## 3. Results

### 3.1. Caspase-6 Is Partially Required to Defend against HSV-1 Skin Infection

Caspase-6 is categorized as an apoptotic executioner during apoptosis which mediates nuclear shrinkage and DNA fragmentation [46,47]. However, caspase-6 is often dispensable for apoptosis [48] and is not required for development since *Casp6*^–/–^ mice are viable and appear normal. Still, caspase-6 can promote diverse modes of cell death, including apoptosis, pyroptosis, and necroptosis [49]. The substrates of caspase-6 include microtubule-associated proteins and α-tubulin [50,51]. Caspase-6 is expressed in neurons [52,53], and caspase-6 has been proposed to be involved in microtubule integrity in axons [51]. Interestingly, caspase-6 is also implicated in neural degenerative diseases and axon pruning [54,55,56,57], which is the process of trimming unnecessary neural connections during neurogenesis [58]. Neurotropic viruses, such as HSV-1, infect neurons and are transported along axon microtubules to establish infection and spread to new cells [59]. Viral infection induces neuronal pruning [60] and axon degeneration [61], which may be a host defense mechanism to prevent viral spread. Thus, we hypothesized that HSV-1-infected neurons undergo caspase-6-mediated axon pruning to limit HSV-1 trafficking. 

**Figure 1:**
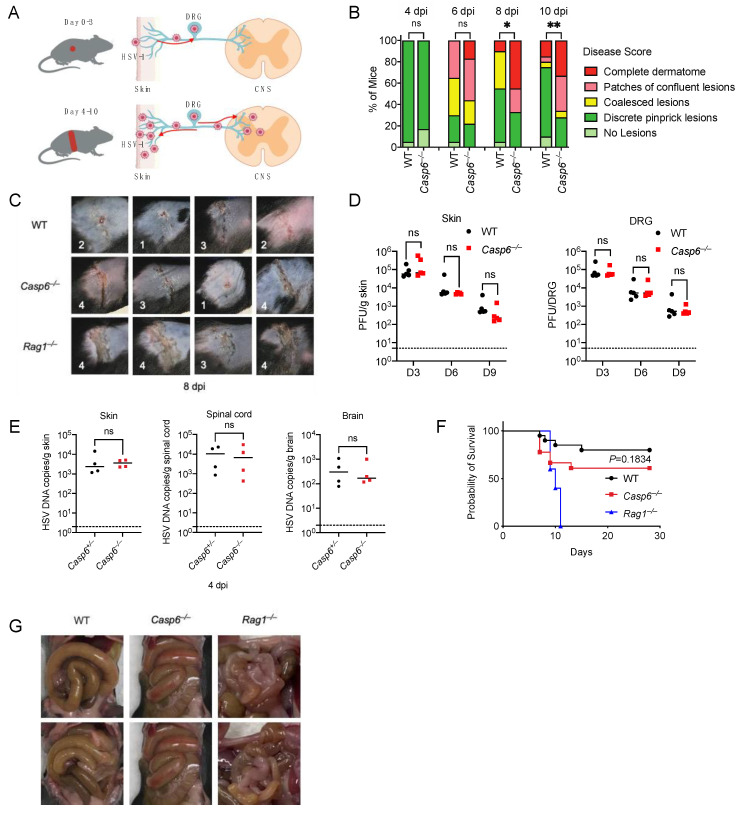
Caspase-6 plays a modest role in defending against HSV-1 skin infection. Mice were infected on the right flank with 10^5^ PFU HSV-1 strain NS. (**A**) Model for HSV-1 mouse flank infection. (**B**) Disease score at day 4, 6, 8, and 10 post-infection (dpi) between WT (*n* = 20) and *Casp6*^–/–^ (*n* = 18) mice. (**C**) Representative skin lesion images at 8 dpi of WT, *Casp6*^–/–^, and *Rag1*^–/–^ mice. Disease scores are annotated on each image. (**D**) HSV-1 titers in skin and DRG of WT (*n* = 5, 5, 5 for 3, 6, 9 dpi) and *Casp6*^–/–^ (*n* = 5, 5, 5 for 3, 6, 9 dpi) mice at 3, 6, and 9 dpi were determined by plaque assay on Vero cells. (**E**) HSV-1 DNA copies were determined at 4 dpi in the skin, spinal cord, and brain of littermate control of *Casp6*^+/–^ (*n* = 4) and *Casp6*^–/–^ (*n* = 4) mice by qPCR. (**F**) Survival was monitored between WT (*n* = 20), *Casp6*^–/–^ (*n* = 18) and *Rag1*^–/–^ (*n* = 5) mice. (**G**) Representative intestine images of moribund WT, *Casp6*^–/–^, and *Rag1*^–/–^ mice. Data are representative of three (**B**,**G**) or two experiments (**F**). Dashed lines indicate detection limit. ns: not significant; * *p*  <  0.05; ** *p*  <  0.01.

To test this, we infected wild-type (WT) mice and *Casp6*^–/–^ mice with HSV-1 on the right flank (Figure 1A). The infected mice were monitored for skin zosteriform lesions and survival. Skin lesion severity was recorded as previously described [7,39,40]. *Casp6*^–/–^ mice developed more severe skin lesions than WT mice (Figure 1B,C), especially on days 8 and 10 post-infection (dpi). *Rag1*^–/–^ mice were used as a positive control as they lack adaptive immunity and are susceptible to HSV-1 skin infection [62]. *Rag1*^–/–^ mice had much more severe skin lesions than WT mice (Figure 1C). We measured viral titers in the skin and dorsal root ganglia (DRG) on 3, 6, and 9 dpi using plaque assays. Surprisingly, we found similar viral titers between WT and *Casp6*^–/–^ mice at all time points examined (Figure 1D). To minimize the effect of the microbiota, we infected littermate control *Casp6*^+/–^ and *Casp6*^–/–^ mice, and then compared viral loads in the skin, spinal cord, and brain at 4 dpi using qPCR. We found no significant differences in viral burdens (Figure 1E). We then monitored survival in WT and *Casp6*^–/–^ mice. Although *Casp6*^–/–^ mice exhibited a higher lethality rate compared to WT mice (39% versus 20%), this difference did not reach statistical significance (*p* = 0.18, log-rank test). As a positive control, *Rag1*^–/–^ mice were 100% susceptible to the infection (Figure 1F; Table S1). Notably, the moribund WT and *Casp6*^–/–^ mice all had enlarged intestines (Figure 1G), consistent with previous publications in WT mice [62,63], where it was speculated that DRG infection resulted in descending infection from the DRG to the intestines as well as the skin. Interestingly, although *Rag1*^–/–^ mice were susceptible to infection, most of them developed less severe intestinal disorders than WT mice (Figure 1G), suggesting that adaptive immune cells not only control HSV-1 infection but also contribute to intestinal pathology in addition to previously described contributions of myeloid cell activity [64,65]. Alternatively, *Rag1*^–/–^ mice may have succumbed to HSV-1 infection before developing intestinal symptoms. Altogether our results suggest that caspase-6 plays a modest role in innate defense against HSV-1 skin infection.

### 3.2. Caspase-6 Is Partially Required to Control HSV-1 Footpad Infection 

**Figure 2:**
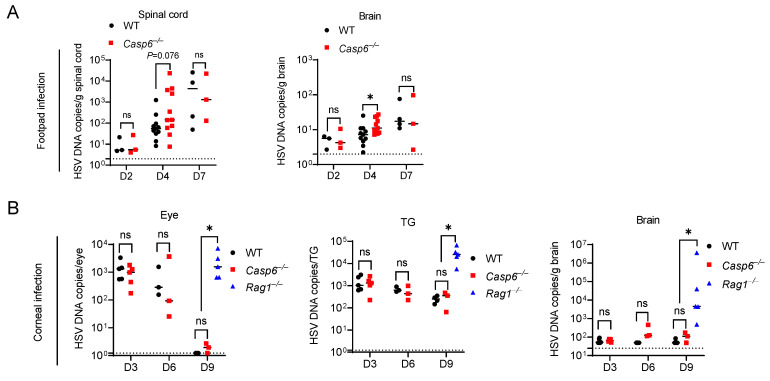
Caspase-6 is partially required to control HSV-1 footpad infection and is not required to control HSV-1 corneal infection. (**A**) WT (*n* = 3, 11, 4 for 2, 4, 7 dpi) and *Casp6*^–/–^ (*n* = 3, 11, 3 for 2, 4, 7 dpi) mice were infected with 10^4^ PFU HSV-1 strain NS via footpad injection. HSV-1 DNA copies were determined at 2, 4, and 7 dpi in the spinal cord and brain of WT and *Casp6*^–/–^ mice by qPCR. (**B**) WT (*n* = 5, 3, 4 for 3, 6, 9 dpi), *Casp6*^–/–^ (*n* = 5, 3, 3 for 3, 6, 9 dpi), and *Rag1*^–/–^ (*n* = 5 for 9 dpi) mice were infected with 10^6^ PFU HSV-1 strain F via corneal infection. HSV-1 DNA copies were determined in the eye, TG, and brain of WT and *Casp6*^–/–^ mice at 3, 6, and 9 dpi and *Rag1*^–/–^ mice at 9 dpi by qPCR. Dashed lines indicate detection limit. ns: not significant; * *p*  <  0.05.

The type of neuron targeted for infection and the length of those axons may influence the outcome of neurotropic viral infection [59,66]. To test the role of caspase-6 more thoroughly on HSV-1 infection, we used the footpad infection model. In this model, following footpad injection, HSV-1 replicates in epithelial cells before it spreads via the sciatic nerve to the CNS [10]. The sciatic nerve is the largest nerve in mice [67]; therefore, the role of caspase-6-mediated axon pruning in limiting HSV-1 trafficking may be enhanced as axonal length lengthens. Following injection of HSV-1 into the footpad, we observed a similar extent of footpad swelling in WT and *Casp6*^–/–^ mice. We then determined HSV-1 burdens in the spinal cord and brain between WT and *Casp6*^–/–^ mice at 2, 4, and 7 dpi. Viral loads increased in WT and *Casp6*^–/–^ mice over time (Figure 2A). *Casp6*^–/–^ mice had significantly higher viral burdens in the brain at 4 dpi compared to WT mice. There was also a trend of increased viral burdens in the spinal cord of *Casp6*^–/–^ mice at 4 dpi that did not reach statistical significance (*p*  =  0.076). However, there were no significant differences at 2 or 7 dpi (Figure 2A), although we used lower numbers of mice in these timepoints because we wanted to maximize experimental power at 4 dpi. Therefore, we conclude that caspase-6 reduces HSV-1 burdens in the CNS at certain time points. Although other interpretations are possible, the restriction of HSV-1 by caspase-6 could be due to an intrinsic inhibition of viral trafficking in neurons.

### 3.3. Caspase-6 Is Dispensable for Control of HSV-1 Corneal Infection

We next used a mouse corneal infection model to examine the role of caspase-6 in HSV-1 corneal infection. In this model, HSV-1 first infects the corneal epithelium and then spreads via the ciliary and ophthalmic nerves to the trigeminal ganglion (TG) and brain [11,12]. Following the corneal inoculation of WT and *Casp6*^–/–^ mice, we measured HSV-1 burdens in the eye, TG, and brain at 3, 6, and 9 dpi. There were no significant differences between WT and *Casp6*^–/–^ mice at all time points examined (Figure 2B). Thus, we concluded that caspase-6 does not contribute to the defense against HSV-1 corneal infection.

### 3.4. Caspase-6 Is Not Required to Defend against HSV-1 ΔUs3 Strain Infection

**Figure 3:**
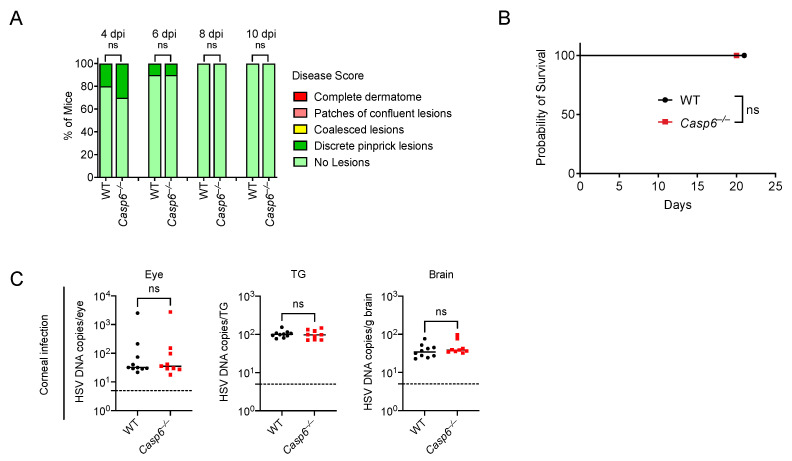
Caspase-6 is dispensable to control HSV-1 Δ*Us3* infection. (**A**,**B**) WT (*n* = 10) and *Casp6*^–/–^ (*n* = 10) mice were infected with 10^6^ HSV-1 Δ*Us3* strain via skin inoculation. Disease scores (**A**) and survival (**B**) were monitored daily between WT and *Casp6*^–/–^ mice. (**C**) WT (*n* = 10) and *Casp6*^–/–^ (*n* = 9) mice were infected with 10^6^ HSV-1 Δ*Us3* strain via ocular inoculation. HSV-1 DNA copies were determined in the eye, TG, and brain of WT and *Casp6*^–/–^ mice at 4 dpi by qPCR. Data are pooled from two experiments (**A**–**C**). Dashed lines indicate detection limit. ns: not significant.

As a successful host-adapted pathogen, HSV-1 can suppress host immune responses. HSV-1 encodes a protein called US3 that inhibits apoptosis [68,69,70,71] and an HSV-1 Δ*Us3* mutant induces more apoptosis in vivo than WT HSV-1 [72,73,74]. Since we observed only a modest role of caspase-6 in defending against WT HSV-1, we reasoned that the activation of caspase-6 might be inhibited by HSV-1. Therefore, an apoptosis-inducing HSV-1 strain may accentuate the role of caspase-6 during infection. We first used the skin infection model and infected WT and *Casp6*^–/–^ mice with the HSV-1 Δ*Us3* strain. We observed that the HSV-1 Δ*Us3* strain induced minimal skin lesions in both WT and *Casp6*^–/–^ mice, all of which survived the infection (Figure 3A,B; Table S1). We then used a corneal infection model with WT and *Casp6*^–/–^ mice. After infection of the HSV-1 Δ*Us3* strain in the cornea, we measured HSV-1 burdens in the eye, TG, and brain at 4 dpi. There were no significant differences in viral burdens between WT and *Casp6*^–/–^ mice (Figure 3C). The viral burdens were lower than in mice infected with the HSV-1 WT strain (Figure 2B), which is consistent with a previous publication showing that the HSV-1 Δ*Us3* strain is less infectious in vivo than the HSV-1 WT strain [75]. 

### 3.5. Caspase-7 Is Not Required to Defend against HSV-1 Skin Infection

**Figure 4:**
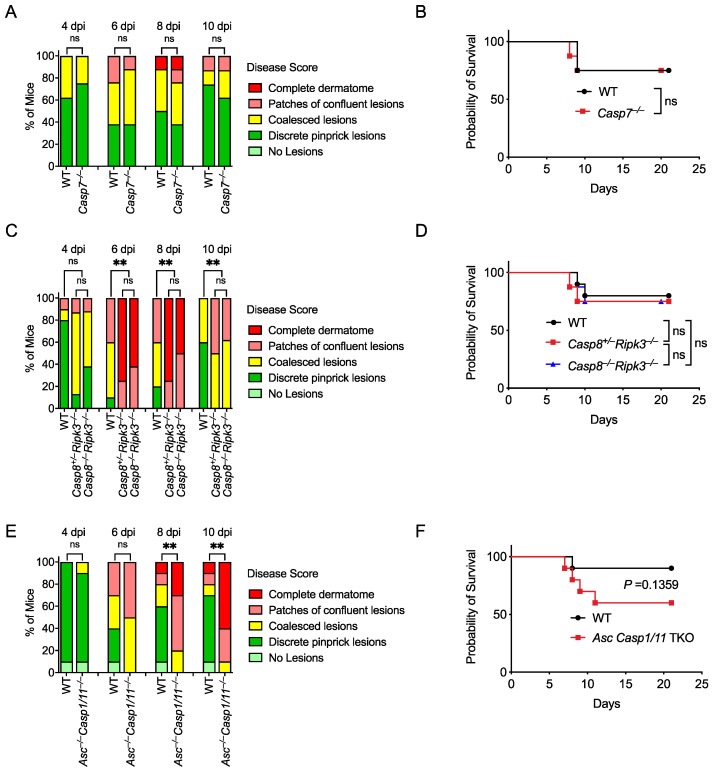
Caspase-7 and caspase-8 are not required to control HSV-1 skin infection, while ASC is protective in the absence of inflammatory caspases. Mice were infected at the right flank with 10^5^ PFU HSV-1 strain NS. Disease scores (**A**,**C**,**E**) and survival (**B**,**D**,**F**) were monitored between WT and knockout mice. Mice number in: (**A**,**B**) WT *n* = 8, *Casp7*^–/–^ *n* = 8; (**C**,**D**) WT *n* = 10, *Casp8*^+/–^*Ripk3*^–/–^ *n* = 8, *Casp8*^–/–^*Ripk3*^–/–^ *n* = 8; (**E**,**F**) WT *n* = 10, *Asc^–/–^Casp1/11^–/–^ n* = 10. Data are pooled from two experiments (**A**–**F**). ns: not significant; ** *p*  <  0.01.

Perforin and subsequent apoptosis induction are essential for successful adaptive immune responses against HSV-1 infection [76,77]. Caspase-7 had been considered a poor backup for caspase-3 to execute apoptosis; however, our laboratory has recently revealed a unique function of caspase-7 in repairing plasma membrane pores that are caused by GSDMD or perforin [17]. We found that *Casp7*^–/–^ mice and *Prf1*^–/–^ mice showed similar susceptibility to *Chromobacterium violaceum* and *Listeria monocytogenes* [17]. Therefore, we investigated whether caspase-7 was also required for defense against HSV-1 skin infection. However, skin lesion severity and survival were similar between WT and *Casp7*^–/–^ mice (Figure 4A,B; Table S1). Thus, caspase-7 is not required to control HSV-1 skin infection.

### 3.6. Caspase-8 Is Not Required for Defense against HSV-1, but Ripk3 Is Important

Caspase-8 is an apoptotic initiator in the extrinsic apoptosis pathway that is activated in response to death receptor activation [78]. We next investigated whether caspase-8 is involved in HSV-1 skin infection. Since *Casp8*^–/–^ mice are embryonically lethal due to unchecked necroptosis that is executed by the RIPK3-MLKL axis [33,79], we compared WT and *Casp8*^+/–^*Ripk3*^–/–^ with *Casp8*^–/–^*Ripk3*^–/–^ mice during HSV-1 skin infection to assess the role of caspase-8. Skin lesions and survival were similar between *Casp8*^+/–^*Ripk3*^–/–^ and *Casp8*^–/–^*Ripk3*^–/–^ mice, suggesting caspase-8 is not involved in defense against HSV-1 infection (Figure 4C,D; Table S1).

HSV-1 inhibits necroptosis in human cells using ICP6, whose RHIM domain interferes with the RHIM–RHIM interaction between RIPK1 and RIPK3 in the TNFR1 signaling pathway [80]. However, in mouse cells, this virulence effect backfires on the species-mismatched virus, as the ICP6 RHIM domain now causes murine RIPK3 to activate, resulting in necroptosis that is beneficial to the mouse [81,82]. Thus, mice deficient in the necroptosis pathway are more susceptible to HSV-1 infection via intravenous or intraperitoneal injections [81,82]. We used the skin inoculation route and found that RIPK3-deficient mice (*Casp8*^+/–^*Ripk3*^–/–^) developed more severe skin lesions compared with WT mice (Figure 4C), suggesting that RIPK3-mediated necroptosis is protective against HSV-1 skin infection, in agreement with its role after systemic inoculation. This protective effect of RIPK3 against skin lesion severity did not translate into a difference in survival as there was no significant difference between WT, *Casp8*^+/–^*Ripk3*^–/–^, and *Casp8*^–/–^*Ripk3*^–/–^ mice (Figure 4D, Table S1). This discordance indicates that skin lesion differences are not always associated with differences in survival, in contrast to prior studies using intraperitoneal and intravenous inoculation routes, where survival differences were observed [81,82].

### 3.7. Asc^–/–^Casp1/11^–/–^ Mice Develop More Severe Skin Lesions

Inflammasomes are molecular complexes that include a sensor and an adaptor protein called apoptosis-associated speck-like protein containing a CARD (ASC) that mediates caspase-1 activation. Caspase-1 processes proinflammatory cytokines and induces a lytic form of cell death called pyroptosis. There are multiple inflammasomes that activate caspase-1, including NLRP3 [83], AIM2 [84], and IFI16 [85], which have been shown to detect HSV-1. HSV-1 induces inflammasome activation in multiple cell types, including keratinocytes [84], fibroblasts [85], and macrophages [86]. The other inflammatory caspase, caspase-11, detects the presence of bacterial lipopolysaccharide in the cytosol of host cells. Because *Casp1* and *Casp11* are encoded adjacent to each other and the original *Casp1*^–/–^ mice were contaminated by a passenger mutation in *Casp11* [87], *Casp1/11*^–/–^ mice are often used. *Casp1/11*^–/–^ mice have previously been published to have normal survival after HSV-1 skin infection [63]. However, *Nlrp3*^–/–^ mice have worse pathology after corneal infection [83]. We examined mice that lacked both these caspases and the adaptor protein ASC, which can also activate caspase-8 in the absence of caspase-1 [78,88,89]. Interestingly, *Asc*^–/–^*Casp1/11*^–/–^ mice developed more severe zosteriform lesions and trended towards lower survival than WT mice (Figure 4E,F; Table S1). This could indicate that caspase-8 is redundant with caspase-1 in reducing the severity of HSV-1 infection. However, more evidence is needed to conclude whether the ASC-caspase-8 pathway drives this phenotype.

### 3.8. Caspase-14 and Gasdermin A Are Not Required for Defense against HSV-1 Skin Infection

We next considered two cell death genes, caspase-14 and GSDMA, that share a specific expression pattern in stratified epithelial cells, such as keratinocytes of the skin [18,19,28]. Keratinocytes are a major target cell type for HSV-1 replication during primary and recurrent infection. Interestingly, human papillomavirus 8 blocks caspase-14 activation in keratinocytes [90], suggesting that caspase-14 may have some antiviral functions. Caspase-14 can be activated by an epidermal serine protease called Kallikrein-related peptidase-7 [91]. To our knowledge, caspase-14 activation has not been studied in the context of skin infections.

**Figure 5:**
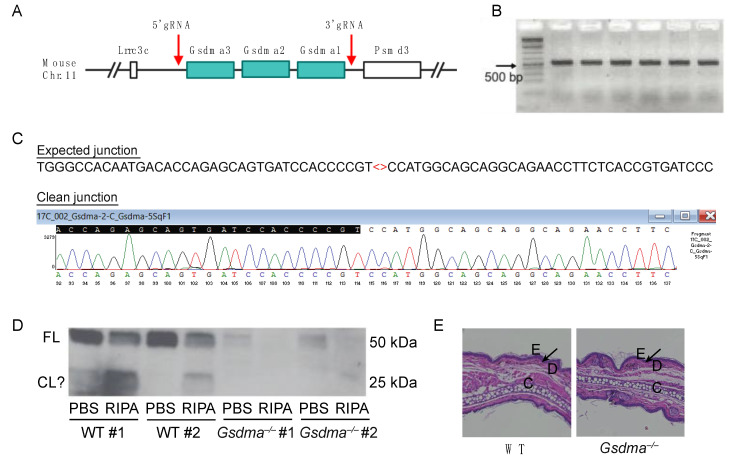
Generation of *Gsdma1-Gsdma2-Gsdma3* triple knockout (*Gsdma*^–/–^) mice. (**A**) *Gsdma1*, *Gsdma2*, and *Gsdma3* are clustered on mouse chromosome 11. CRISPR was used to delete all three genes at the red arrows. (**B**) *Gsdma*^–/–^ founder mice were validated by PCR using the primer sets (Gsdma-5ScF1 and Gsdma-3ScR1) described in Materials and Methods. (**C**) *Gsdma*^–/–^ founder mice were verified by Sanger Sequencing, showing a 50kb deletion. (**D**) Western blot of GSDMA using ear tissues of WT and *Gsdma*^–/–^ mice extracted by PBS and RIPA buffer. FL, full-length GSDMA; CL?, potential cleavage products of GSDMA. (**E**) Histological staining of ear sections from WT and *Gsdma*^–/–^ mice. E, epidermis; D, dermis; C, cartilage. The epidermal–dermal junctions are indicated by the black arrows.

Humans encode a single GSDMA gene, whereas this is triplicated in mice to three adjacent genes, *Gsdma1*, *Gsdma2*, and *Gsdma3* (Figure 5A). GSDMA can be cleaved by the *Streptococcus* protease SpeB, but a host protease cleaving GSDMA has not yet been identified. To examine the function of GSDMA genes in mice, we used CRISPR/Cas9 gene editing to generate *Gsdma1-Gsdma2-Gsdma3* triple knockout mice (herein referred to as *Gsdma*^–/–^, Figure 5A). The CRISPR deletion was verified by PCR (Figure 5B), and the clean junction was confirmed by Sanger Sequencing (Figure 5C). Western blot confirmed the absence of GSDMA expression in the skin tissue of *Gsdma*^–/–^ mice (Figure 5D). In the skin tissue of WT mice, GSDMA was present in PBS and RIPA buffer extracts, suggesting that GSDMA is present both in the cytosol and is associated with the membrane compartment. Interestingly, potentially cleaved GSDMA fragments of ~25 kDa were present in WT tissue. However, it is difficult to determine whether these cleavage products are present in naïve mice or due to experimental artifacts. In our colony, *Gsdma*^–/–^ mice appeared healthy with no signs of developmental defects, which was supported by the normal skin structure of *Gsdma*^–/–^ mice compared to naïve WT mice (Figure 5E).

**Figure 6:**
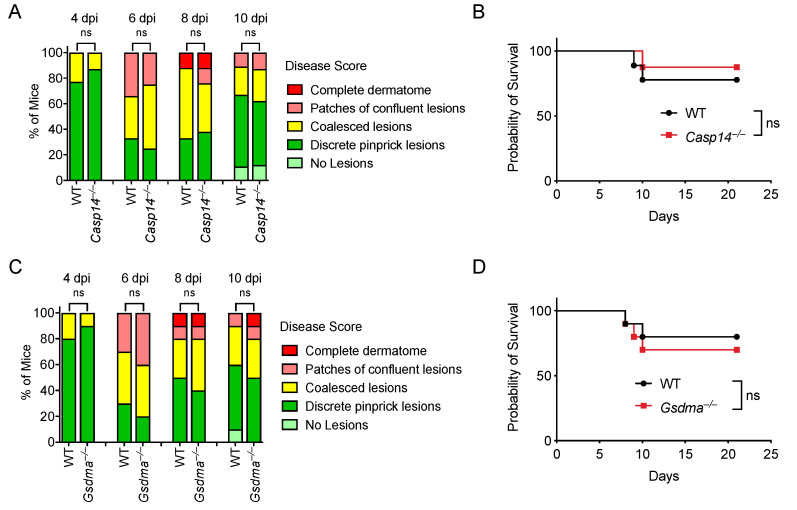
Caspase-14 and GSDMA are not required to control HSV-1 skin infection. Mice were infected on the right flank with 10^5^ PFU HSV-1 strain NS. Disease scores (**A**,**C**) and survival (**B**,**D**) were monitored between WT and knockout mice. Mice number in: (**A**,**B**) WT *n* = 9, *Casp14*^–/–^ *n* = 8; (**C**,**D**) WT *n* = 10, *Gsdma*^–/–^ *n* = 10. Data are pooled from two experiments (**A**–**D**). ns: not significant.

Given that (1) most gasdermins can be activated by a caspase [92], (2) caspase-14 is an enigmatic caspase with undefined substrates, (3) no known host proteases have been identified to activate GSDMA, and (4) both caspase-14 and GSDMA are highly expressed in keratinocytes, we hypothesized that caspase-14 activates GSDMA in keratinocytes to facilitate viral clearance during HSV-1 infection. We evaluated *Casp14*^–/–^ and *Gsdma*^–/–^ mice during HSV-1 skin infection. However, WT, *Casp14*^–/–^, and *Gsdma*^–/–^ mice developed similar skin lesions during HSV-1 infection (Figure 6A,C). Similarly, *Casp14*^–/–^ and *Gsdma*^–/–^ mice had similar survival rates compared to WT mice (Figure 6B,D; Table S1). Therefore, caspase-14 and GSDMA are not required for defense against HSV-1 skin infection.

## 4. Discussion

Regulated cell death, including apoptosis, pyroptosis, and necroptosis, plays important role in defending against infection. As an apoptosis executioner and axon pruning mediator, caspase-6 is partially required to control HSV-1 infection, as shown by increased skin lesions and higher brain viral burdens in *Casp6*^–/–^ mice. One interpretation is that caspase-6 is activated by HSV-1 infection in neurons to restrict viral trafficking via axon degeneration. However, since HSV-1 infects multiple cell types in these models, it is possible that caspase-6 functions in keratinocytes or other cell types to defend against HSV-1. Further studies are needed to determine the activation status of caspase-6 in different cell types in vivo. The phenotypes we observed in *Casp6*^–/–^ mice were modest. This could be due to viral virulence factors blocking apoptosis at multiple steps [93]. Thus, we tested HSV-1 *ΔUs3* strain, which was shown to induce more apoptosis [72,73,74]. Although the mutant virus is less virulent than its WT counterpart, *Casp6*^–/–^ mice showed equal skin lesions, survival, and viral burdens compared to WT mice. Since HSV-1 can inhibit apoptosis at multiple steps using a battery of viral proteins [93], it is possible that other viral proteins (e.g., ICP22, ICP27 [22,94], and US5 [95,96]) are redundant at inhibiting caspase-6 activation. Therefore, other apoptosis-inducing HSV-1 strains can be tested in *Casp6*^–/–^ mice to assess if there will be a stronger phenotype. Another neurotropic virus, Theiler’s murine encephalomyelitis virus (TMEV), was shown to induce axon degeneration to prevent viral transportation in mice [61]. Therefore, it is worthwhile to test whether *Casp6*^–/–^ mice are sensitive to TMEV. There are other neurotropic viruses that would be interesting to test in *Casp6*^–/–^ mice, such as pseudorabies virus, West Nile virus, encephalomyocarditis virus, and mouse hepatitis virus.

Another two apoptotic caspases, caspase-7 and -8, were not required to control HSV-1 skin infection. However, both caspase-7 and -8 can be inhibited by HSV-1. For example, HSV-1 latency-associated transcript (LAT) blocks caspase-8 activity in neurons [97,98]. Thus, future studies need to investigate apoptosis-inducing HSV-1 strains to reveal whether caspase-7 and -8 could function in defense against HSV-1 in the absence of their inhibitions.

The role of inflammatory caspases during HSV-1 infection requires further investigation. HSV-1 activates multiple inflammasomes [83,85,99,100]; however, HSV-1 also inhibits inflammasome activation [85,101]. Caspase-1 and its downstream molecules, IL-1β and IL-18, have been shown to be either protective [42,83,102,103,104,105,106] or detrimental [107,108] during HSV-1 infections, depending on different models. *Casp1/11*^–/–^ mice were previously published as having skin lesions similar to WT mice after HSV-1 skin infection [63]; however, our data showed that the *Asc^–/–^Casp1/11^–/–^* mice are more susceptible. This could be due to ASC mediating an inflammasome-independent function to defend against HSV-1 infection. ASC can signal to caspase-8 and trigger apoptosis [78,88,89], thus inflammasome-mediated pyroptosis and caspase-8-mediated apoptosis may be redundant in defending against HSV-1 infection. Future studies are needed to determine which cell type ASC functions and the role of ASC in the absence of inflammatory caspases in HSV-1 infection.

The role of gasdermins in HSV-1 infection is starting to be revealed. HSV-1 induces GSDMD-mediated pyroptosis in macrophages [109] and GSDME-mediated pyroptosis in human keratinocytes [110]. Our initial hypothesis was that caspase-14 activates GSMDA in keratinocytes to defend against HSV-1 infection. Although our data suggested that caspase-14 and GSDMA are not involved in defending against HSV-1 infection, it is likely that the function of caspase-14 or GSDMA in keratinocytes is masked by HSV-1-encoded proteins, because a recent study showed that HSV-1 *ΔICP27* strain but not WT HSV-1 induces GSDME-mediated pyroptosis in human keratinocytes [110]. Thus, future studies that evaluate additional mutant HSV-1 strains could unmask a possible role for caspase-14 and GSDMA during viral infection. Moreover, the activation of both caspase-14 and GSDMA needs further investigation. Determining whether activated caspase-14 cleaves GSDMA in an in vitro system will provide valuable information. However, such studies are complicated by the lack of a system to obtain activated caspase-14 in vitro.

In summary, our data highlight HSV-1 as a successful host-adapted pathogen. The lack of strong phenotypes suggests that using mutant viral strains could reveal the function of host proteins that may be otherwise masked by viral inhibitory proteins.

## Data Availability

Not applicable.

## Supplementary Materials

The following supporting information can be downloaded at: https://www.mdpi.com/article/10.3390/v14092034/s1, Table S1. Survival table of HSV-1 flank infected mice.
