# Supplementary material for: Role of Caspases and Gasdermin A during HSV-1 Infection in Mice
Source: Viruses. 2022 Sep 13;14(9):2034. doi: 10.3390/v14092034 (PMC9504851; doi:10.3390/v14092034)
Supplement: Supplementary file 1 [file viruses-14-02034-s001.zip › viruses-1899986-supplementary.pdf]

Survival on:	6 dpi	7 dpi	8 dpi	9 dpi	10 dpi	11 dpi	12 dpi	13 dpi	14 dpi	15 dpi	Survived	Corresponding Figures
**Mice**												
**WT**	20/20	19/20	18/20	18/20	17/20	17/20	17/20	17/20	17/20	16/20	16/20	Figure 1F
Casp6$^{-/-}$	18/18	14/18	14/18	12/18	12/18	12/18	12/18	11/18	11/18	11/18	11/18	
Rag1$^{-/-}$	5/5	5/5	5/5	3/5	2/5	0/5	0/5	0/5	0/5	0/5	0/5	
**WT**	10/10	10/10	10/10	10/10	10/10	10/10	10/10	10/10	10/10	10/10	10/10	Figure 3B
Casp6$^{-/-}$	10/10	10/10	10/10	10/10	10/10	10/10	10/10	10/10	10/10	10/10	10/10	
**WT**	8/8	8/8	8/8	6/8	6/8	6/8	6/8	6/8	6/8	6/8	6/8	Figure 4B
Casp7$^{-/-}$	8/8	8/8	7/8	6/8	6/8	6/8	6/8	6/8	6/8	6/8	6/8	
**WT**	10/10	10/10	10/10	9/10	8/10	8/10	8/10	8/10	8/10	8/10	8/10	Figure 4D
Casp8$^{+/-}$Ripk3$^{-/-}$	8/8	8/8	7/8	6/8	6/8	6/8	6/8	6/8	6/8	6/8	6/8	
Casp8$^{-/-}$Ripk3$^{-/-}$	8/8	8/8	7/8	7/8	6/8	6/8	6/8	6/8	6/8	6/8	6/8	
**WT**	10/10	10/10	9/10	9/10	9/10	9/10	9/10	9/10	9/10	9/10	9/10	Figure 4F
Asc$^{-/-}$Casp1/11$^{-/-}$	10/10	9/10	8/10	7/10	7/10	6/10	6/10	6/10	6/10	6/10	6/10	
**WT**	9/9	9/9	9/9	8/9	7/9	7/9	7/9	7/9	7/9	7/9	7/9	Figure 6B
Casp14$^{-/-}$	8/8	8/8	8/8	8/8	7/8	7/8	7/8	7/8	7/8	7/8	7/8	
**WT**	10/10	10/10	9/10	9/10	8/10	8/10	8/10	8/10	8/10	8/10	8/10	Figure 6D
Gsdma$^{-/-}$	10/10	10/10	9/10	8/10	7/10	7/10	7/10	7/10	7/10	7/10	7/10	

Supplementary Table S1. Survival table of HSV-1 flank infected mice. Mice were infected on the right flank with 10^5 PFU HSV-1 strain NS (Figures 1F, 4B, 4D, 4F, 6B, and 6D) or with 10^6 PFU HSV-1 *ΔUs3* strain (Figure 3B). Survival was monitored daily until at least 21 dpi. dpi, day post-infection.
